# Evaluation of Two Highly-Multiplexed Custom Panels for Massively Parallel Semiconductor Sequencing on Paraffin DNA

**DOI:** 10.1371/journal.pone.0128818

**Published:** 2015-06-03

**Authors:** Vassiliki Kotoula, Aggeliki Lyberopoulou, Kyriaki Papadopoulou, Elpida Charalambous, Zoi Alexopoulou, Chryssa Gakou, Sotiris Lakis, Eleftheria Tsolaki, Konstantinos Lilakos, George Fountzilas

**Affiliations:** 1 Laboratory of Molecular Oncology, Hellenic Foundation for Cancer Research, Aristotle University of Thessaloniki School of Medicine, Thessaloniki, Greece; 2 Department of Pathology, Aristotle University of Thessaloniki School of Medicine, Thessaloniki, Greece; 3 Health Data Specialists Ltd, Athens, Greece; 4 Department of Haematology, “Laikon” General Hospital, University of Athens Medical School, Athens, Greece; NIDCR/NIH, UNITED STATES

## Abstract

**Background—Aim:**

Massively parallel sequencing (MPS) holds promise for expanding cancer translational research and diagnostics. As yet, it has been applied on paraffin DNA (FFPE) with commercially available highly multiplexed gene panels (100s of DNA targets), while custom panels of low multiplexing are used for re-sequencing. Here, we evaluated the performance of two highly multiplexed custom panels on FFPE DNA.

**Methods:**

Two custom multiplex amplification panels (B, 373 amplicons; T, 286 amplicons) were coupled with semiconductor sequencing on DNA samples from FFPE breast tumors and matched peripheral blood samples (n samples: 316; n libraries: 332). The two panels shared 37% DNA targets (common or shifted amplicons). Panel performance was evaluated in paired sample groups and quartets of libraries, where possible.

**Results:**

Amplicon read ratios yielded similar patterns per gene with the same panel in FFPE and blood samples; however, performance of common amplicons differed between panels (p<0.001). FFPE genotypes were compared for 1267 coding and non-coding variant replicates, 999 out of which (78.8%) were concordant in different paired sample combinations. Variant frequency was highly reproducible (Spearman’s rho 0.959). Repeatedly discordant variants were of high coverage / low frequency (p<0.001). Genotype concordance was (a) high, for intra-run duplicates with the same panel (mean±SD: 97.2±4.7, 95%CI: 94.8–99.7, p<0.001); (b) modest, when the same DNA was analyzed with different panels (mean±SD: 81.1±20.3, 95%CI: 66.1–95.1, p = 0.004); and (c) low, when different DNA samples from the same tumor were compared with the same panel (mean±SD: 59.9±24.0; 95%CI: 43.3–76.5; p = 0.282). Low coverage / low frequency variants were validated with Sanger sequencing even in samples with unfavourable DNA quality.

**Conclusions:**

Custom MPS may yield novel information on genomic alterations, provided that data evaluation is adjusted to tumor tissue FFPE DNA. To this scope, eligibility of all amplicons along with variant coverage and frequency need to be assessed.

## Introduction

The publication of whole genome sequencing data of 1000s of cancer genomes and the affordability of next-generation sequencers for more limited applications have transformed the way tumor genotyping is viewed and performed in recent years. Thus, targeted massively parallel sequencing (MPS), i.e., the simultaneous analysis of 100s of small regions in multiple genes for the identification of selected disease-related genomic variants is progressively replacing classic Sanger sequencing and pyrosequencing. The panel of DNA targets to be analyzed is selected according to research or diagnostic questions. The most frequent applications of such panels are genetic tests for hereditary disease, performed in germline DNA from blood samples, e.g., [1–4]. Enthusiasm for MPS is supported by the fact that it can be applied on formalin-fixed paraffin-embedded (FFPE) DNA [5–8], which is still the main patient material used for tumor genotyping in clinical research and practice. Although questions on such applications in clinical diagnostics still remain to be answered [9], at least in clinical research, targeted MPS on FFPE is increasingly used because of its cost-effectiveness and also because it allows for the detection of tumor-specific mutations with possible clinical relevance that would have not been suspected and addressed with single-target approaches [10–12].

The most widely used platforms for targeted MPS are Illumina MiSeq and Ion Torrent semiconductor sequencers. Semiconductor-based DNA sequencing uses the non-optical detection of a hydrogen ion (H^+^), released during DNA sequencing-by-synthesis. The H^+^ by-product causes a change in pH that is detected by the semiconductor chip and data is displayed as a peak of voltage [13]. Comparisons of Ion Torrent PGM platform with other next-generation sequencers have been described [5, 14, 15], whereas a comparison between PGM and Proton sequencers was recently performed for the non-invasive detection of fetal abnormalities [16]. The majority of NGS platforms include a PCR step at some point, in order to increase target template exposure for sequencing. For semiconductor MPS, enrichment of the DNA areas of interest is accomplished during a PCR-based library preparation from template DNA as low as 10ng, which is sufficient for the poor quality DNA isolated from FFPE specimens [12, 17].

Regarding FFPE MPS in Oncology, semiconductor sequencing has been applied with the commercially available Ampliseq Cancer Hotspot panel targeting actionable mutations in 46 genes using a multiplex 190-amplicon PCR-based target enrichment [6], an extended version targeting 50 genes [18], and an even more enriched version targeting 409 genes for Proton sequencers [11] These panels have been evaluated in different types of materials (DNA from blood, fresh frozen and FFPE tissue, cytology specimens) and are generally appreciated as a useful and cost-efficient means for FFPE sample genotyping [19–21].

More interestingly though, the Ampliseq multiplex design platform is free for custom target multiplexing that allows researchers to investigate genomic areas of interest other than those included in the fixed content cancer panels. This freedom of target choice offers the potential to immensely increase research flexibility by allowing different sets of DNA areas to be investigated in parallel according to the tasks of individual studies that may address issues beyond the so called clinically “actionable” mutations. As yet, however, this approach has attracted little attention with FFPE DNA from routinely processed tissue material, with low [8] or higher multiplexing [22]. The performance of a custom panel with the Illumina platform on FFPE DNA has also been described recently [23].

In this study, we addressed the efficiency and reliability of highly-multiplexed custom panels with semiconductor sequencing on FFPE DNA. For this purpose, we evaluated amplicon performance and genotype reproduciility with two in-house selected multi-gene panels on the same DNA and on different series of matched blood and FFPE DNA samples that were analyzed on a Proton sequencer.

## Materials and Methods

The present is a pilot study in the frame of two large translational research projects on mapping disease and drug related genomic alterations in >2000 tumours from patients with early high-risk breast cancer who had been treated with adjuvant chemotherapy within randomized phase III trials by HeCOG. The clinical studies have been published for HE 10/97, HE 10/00 and HE 10/05 [24–27], while evaluation of the more recent HE 10/08 is ongoing. Written consent had been obtained by the patients for the use of their biologic material for research purposes. The translational study on this material was approved by the Bioethics Committee of the Aristotle University of Thessaloniki School of Medicine (# 77/10 June 2014) and by the Institutional Review Board of Papageorgiou Hospital of Thessaloniki (# 725/10 May 2013). Peripheral blood and routinely processed FFPE tissue material were retrieved from the HeCOG biologic sample repository; tissue blocks had been collected from 1997–2008. The present pilot was undertaken for evaluating panel performance. For this study, matched series of peripheral blood and tumour DNA, as well as matched series of FFPE DNA data obtained upon MPS with the two custom panels were evaluated.

### Custom Ion panel design for targeted MPS

**Fig 1:**
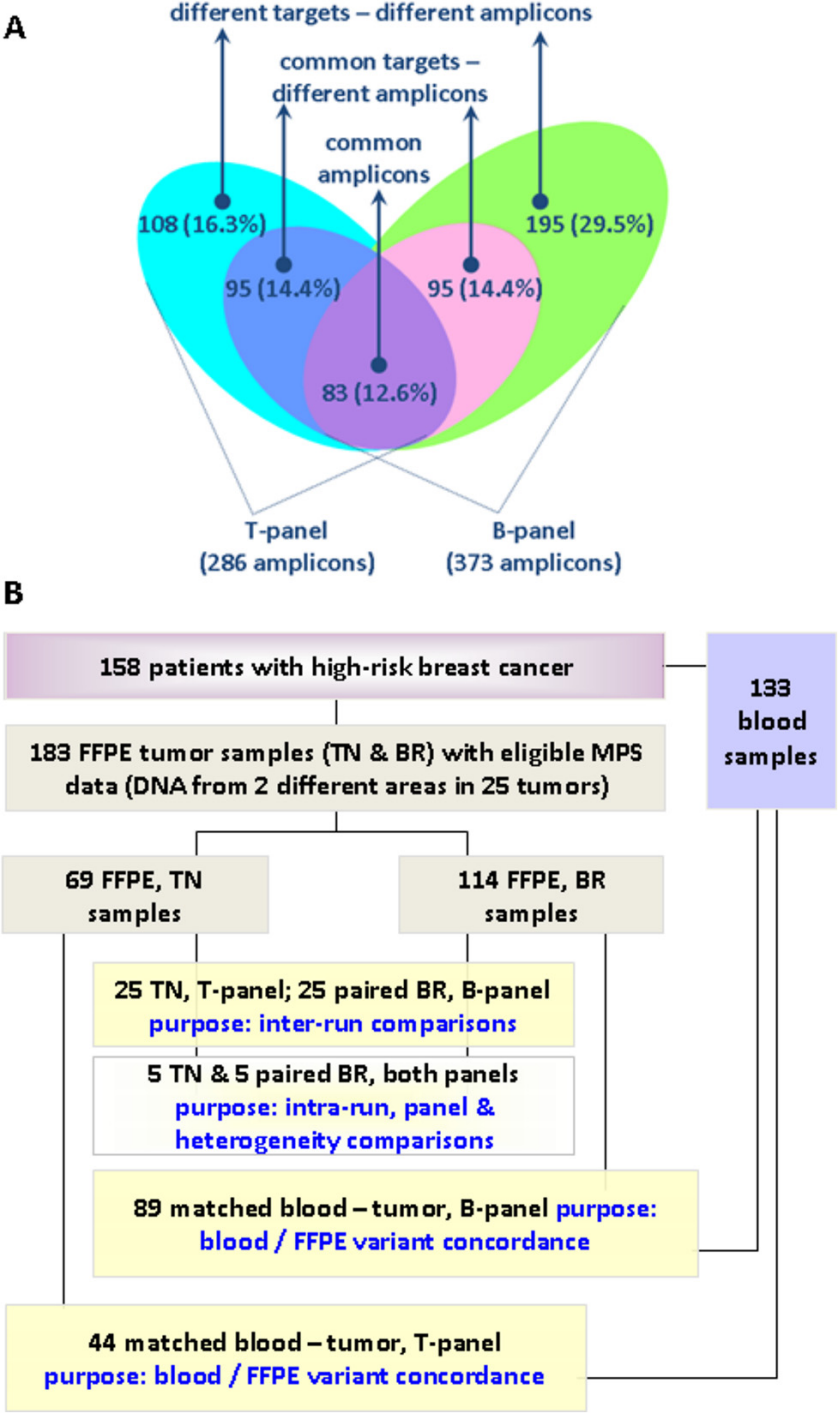
Panel comparison and sample groups. **A.** Comparison of the B and T panels with respect to amplicon targets. Common amplicons had the same ID in both panels. Common target—different amplicons had slightly shifted coordinates targeting the same genomic regions (±10 nts). Percentages among all amplicons in the two panels are shown. **B.** REMARK diagram of patients, samples, and sample groups in this study. In total, 316 DNA samples in 332 libraries were examined.

Custom panels targeting genomic regions and genes previously implicated in TNBC (TNBC-panel [T-panel]) and frequently altered in all breast cancer subtypes, (BREAST panel [B-panel]) were designed according to literature [28–31]. The design was not specifically mutation-oriented and targeted areas in the panels included intron-exon sequences in genes with reported numerical / structural alterations. The T-panel covered a total genomic sequence of ~21Kb with 286 amplicons in 43 genes; in comparison, the B-panel covered ~35Kb with 373 amplicons in 60 genes. The two panels shared 83 common amplicons with the same manufacturing amplicon ID and with identical amplification primer sequences and design region coordinates. Another 95 amplicons with different ID, primer sequences and design start-stop coordinates targeted common DNA regions (±10nts) in the two panels (shifted amplicons). An overview of overlapping targets between the two panels is shown in **Fig 1A**. Detailed sequence data and panel characteristics are shown in **S1 File**.

Panel design was based on the GRCh37 (hg19) genomic reference. Briefly, genomic coordinates for the selected targets were exported from the UCSC Genome Browser, checked for specificity, and submitted to the Ampliseq pipeline through www.ampliseq.com. (Life Technologies / Ion Torrent, Paisley, UK). Amplicon design was adapted for FFPE samples (amplicon length of up to 150bp) and primers were delivered in two pre-mixed pools. Returned primer and amplicon designs were separately evaluated for specificity using the NCBI BLAST tool.

### Tissues and DNA samples

The purpose of this study was not to assess MPS method robustness for obtaining informative results with FFPE samples; thus the T and B panels were evaluated on series of samples that had been classified as eligible for MPS data analysis. Comparisons were undertaken for matched data from 183 FFPE tissue and 133 peripheral blood samples from 158 patients (**Fig 1B**). Amplicon performance was compared for the two panels in (a) 89 cases with matched blood and tumour data for the B-panel; (b) 44 cases with matched blood and tumour data for the T-panel; and, (c) 25 tumours that were tested with both panels on DNA obtained from different areas of the tumour (25 TN samples with the T- and 25 BR samples with the B-panel). Five out of these sample pairs were used for the construction of multiple libraries. Patient demographic and clinicopathologic characteristics of all cases are presented in Tables A—C in **S2 File**.

Total genomic DNA was isolated from peripheral blood lymphocytes following a salt extraction procedure (2-propanol/chloroform/NaCl). DNA was allowed to dissolve for 2 hours at 37°C with TE buffer before quantification [32].

Tissue samples were processed for thorough histologic review, parameter recording and marking as much as possible tumor-dense areas for macro-dissection as described in [33] or tissue microarray construction (TMA). The latter were constructed with 2 X 1.5mm cores from the primary tumor. Tumor cell content (TCC) was assessed as an approximate metric for tumor DNA in the extracted samples, corresponding to tumor nuclei vs. all nuclei in the areas marked for macro-dissection and on the TMA cores. In the present FFPE series, TCC was ≥50% except for 7 samples, for which it was impossible to reach this density, as shown in **S2 File**.

DNA was extracted from TMA cores (5 X 8um thick sections, total depth per block = 0.040mm) or from macro-dissected tissue fragments with magnetic beads (VERSANT Tissue Prep Kit, Siemens Healthcare, Erlangen, Germany). For the (c) series above, TCC was evaluated separately for the two samples from the same tumour. DNA quantity was measured with the Qubit fluorometer (Life Technologies, Paisley, UK) and amplification performance of the template was evaluated by qPCR. Most FFPE samples examined here had ≥2ng/ul DNA amplifiable at Ct≤32 for two different qPCR control assays.

### Sample processing for MPS, data retrieval and analysis

For library construction, 10ng DNA per sample were used as starting material, as per manufacturers instructions. Multiplex PCR was performed using the Ampliseq primer pools with the Ampliseq Library Kit v.2.0 and Ion Xpress barcodes, according to the manufacturer’s instructions (Life Technologies / Ion Torrent, Carlsbad, CA). Library concentration was again normalized to 15ng/ml corresponding to 100pM using Qubit HS DNA kit (Thermo Scientific, Waltham, MA). Clonal template amplification was performed on the Ion Torrent OneTouch-2 instrument followed by enrichment for template Ion Sphere Particles on a One-Touch-ES station. Templating was performed using the Ion PI template OT2-200 Kit, and sequencing was performed on an Ion Proton using PI chips (Ion PI Sequencing 200 Kit v2), with multiplexing up to 96 samples. Run metrics (mapped and on target reads, mean sample read depth, and uniformity) were evaluated in each case (shown for the FFPE tested series in **Tables A—G in S3 File**).

For data retrieval, base calling was performed on the Torrent Server using Torrent Suite v 3.6.2 and v 4.0. Briefly, raw data were transferred to Torrent Server and following signal processing, basecalling is performed and unmapped BAM files are generated. Filtered reads are aligned to the hg19 reference using the TMAP mapper. The TMAP integrates 3 popular alignment algorithms, BWA-short [34] (<150bp), BWA-long [35] (≥150bp), SSAHA [36] (≥150bp), and Super-maximal Exact Matching [37], while it is specifically designed for Ion Torrent products, having a principal error model relating to long homopolymer misscalls mostly resulting in insertion or deletion errors during alignment. After alignment, variant calling was performed with the embedded Variant Caller pipeline (TVC) under high stringency parameters for germline and somatic variant detection. TVC operates on a FreeBayes approach, with minor modifications to allow for Ion Torrent specific error modeling. Reads were visualized on Broad Institute Integrated Genome Viewer for integrity and target alignment. Variants generated from the TVC were uploaded to the Ion Reporter v.4 cloud except for the 44 cases from group (b) and the TN samples in group (c) that had been analyzed with v.1.6 of the same software for further annotation regarding functional effect, presence in dbSNP, COSMIC, ClinVar as well as functional classification based on SIFT, Polyphen and Grantham score.

### Panel amplicon performance evaluation

All samples evaluated here had total coverage, i.e., total amplicon reads >200000 (**Fig A** in **S4 File**). The range of such values, however, was very broad for each sample series, while not all amplicons were read with the same efficiency. E.g., 1000 mapped reads for amplicon X in a sample A with 2000000, and in sample B with 200000 total mapped reads would reflect a higher efficiency for reading the sequence covered by amplicon X in sample B. Therefore, sequencing efficiency was evaluated for amplicon read ratios. These were calculated as amplicon reads vs. total sample reads (% of total reads). Descriptive values of these amplicon read ratios at 10% and 50% (median) were obtained separately for all FFPE samples tested with the B-panel; all blood samples with the B-panel; all FFPE samples with the T-panel; and, all blood samples with the T-panel. In the next, for analysis purposes, we introduced a 4-scale grading for amplicon performance. The cut-off used for amplicon classification was based on the formula 2/N derived from RD/(N x AR), whereby AR (amplicon reads) = 125 is the cut-off for considering eligible amplicons independently of variant presence; RD (read depth) = 250 is the minimum read depth expected for each variant position covered by overlapping amplicons; and, N is the total number of amplicons in the panel. Thus, the minimum acceptable read ratio for the amplicons in the T and B panels was 0.007 and 0.005, respectively. These were applied on the 10^th^ percentile of mean read ratio values that were obtained for all amplicons from all samples. Amplicons with <0.007 for the T panel and <0.005 for the B panel were rated with 0 (failed amplicons); this category practically included amplicons that failed to yield read counts >125 in 90% of the samples in each series. Amplicons with increasing read ratio values up to 1/3 of the series for each panel were rated with 1, indicating marginal performance; all other amplicons were considered of fair performance (grade 2, up to 2/3 of the series, intermediate performing amplicons; grade 3, best performing amplicons). Performance classification for each amplicon with the B and T panels is shown in **Tables A and C in S1 File**.

### Sample eligibility and variant analysis

Steps for excluding ineligible results were originally undertaken in the following order: (i) Raw data for sample metrics (mapped reads, on-target read %, mean depth, read uniformity %), amplicon metrics (number of reads), individual amplicon reads and all variant parameters were merged into the same data file. Amplicon read values <125 were filtered out. (ii) Assessment of sample eligibility: samples with >10% eligible amplicons were considered for analysis. This amplicon-oriented approach was considered more reliable for FFPE samples than the automatically provided mean depth on a sample-centric approach, enforced by observations where samples having sequence metrics on mean depth of >1000 contained subsets of amplicons with coverage of <10. Similarly, the sample Q20 measure did not offer any information on the quality of the obtained results. (iii) Variant eligibility: Low quality variants had automatically been filtered out by Ion Reporter based on embedded filtering metrics. Variants were further excluded for Ion Reporter p value quality metric >0.0001, position coverage <125 *and* variant coverage <50, and if they were non-annotated. Thus, variants at a frequency of 40% in low performing amplicons were accepted (worst case amplicons).

Variant parameters used for panel evaluation included variant annotations for gene, chromosome, coordinate (position), amino acid and coding nucleotide change, dbsnp ID, genotype, coverage, and variant frequency (VF = variant coverage vs. position coverage).

### Confirmatory Sanger sequencing

Orthogonal variant validation by Sanger sequencing was performed for *BRCA1*, *E2F3*, *PIK3CA*, *TP53* and *VEGFA* variants in 50 cases (matched blood—tumor samples). Primer sequences and protocols are described in **Supplemental Methods in S4 File,** while comparisons between Integrated Genome Viewer (IGV) readings and electropherograms are presented in **Figs B and C in S4 File.**

### Statistics

Categorical variables were presented as frequencies and percentages, while various measures (mean, SD, median, min and max) were used for continuous variables. Possible associations between categorical and continuous variables were examined with the Mann-Whitney or Kruskal-Wallis tests, where appropriate. Correlations were calculated using the Pearson’s correlation coefficient or the Spearman’s rank correlation coefficient (Rho). Paired t-test was used for testing the equality of means of paired samples between the different panels, while Wilcoxon singed-rank test for comparing equal distributions of paired samples.

Considering each sample as a separate amplicon-centered dataset the concordance between the two panels was measured for each sample by the use of the Jaccard coefficient. Mean concordance and 95% confidence intervals were computed for each comparison group, while one-sample t-test was used for testing the differentiation from a conservative value of 50.

All variable estimates are presented along with corresponding 95% confidence intervals. All univariate tests were 2-sided with the significance level set at α = 0.05. Contingency tables were created with JMP v.10; descriptive statistics for parameter associations and correlation of continuous variables were performed by using the SPSS v15 and the SAS software for statistical analysis (SAS for Windows, version 9.3, SAS Institute Inc., Cary, NC, USA).

## Results

### Performance of B and T panel amplicons

**Fig 2:**
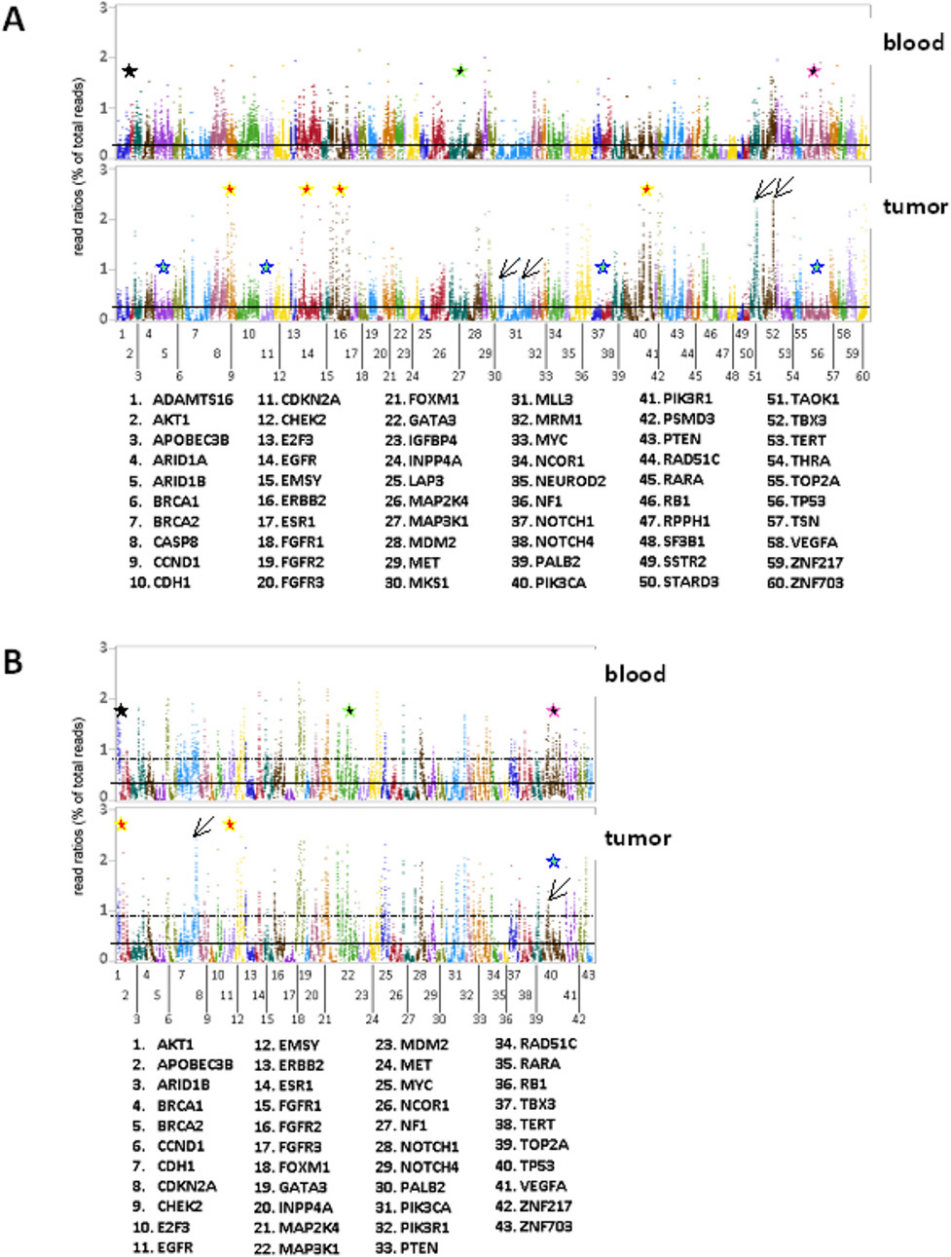
Performance of the B and T panels in DNA from matched blood and tumor FFPE samples. **A:** B panel; **B:** T panel. Read ratios for all amplicons for matched samples sorted per gene are shown, corresponding to 70192 observations with the B and 26128 observations with the T panel. Lanes and dots therein represent mean read ratios per amplicon for all samples tested in the respective group. Amplicon order is the same in all graphs. Solid and dotted horizontal lines within graphs: mean values + 3xSD per panel per sample type, respectively. Amplicon reading efficiency was overall constant between blood—FFPE samples with the same panel. For some genes with frequent gains in breast cancer, e.g., *CCND1*, *EGFR*, *ERBB2*, *PIK3CA* (B panel) and *AKT1*, *EGFR* (T panel) outliers with maximal amplicon read ratios outside the Y-axis were observed (A & B, red stars in tumor graphs). By contrast, for genes frequently lost in breast cancer, e.g., TP53 with both panels, mean read ratios in tumor DNA were lower than in blood (A & B, turquoise stars). FFPE-specific over-representation of individual amplicons was occasionally observed (A & B, diagonal arrows). Importantly, patterns of read ratios occasionally differed for genes targeted with the same amplicons in panels B and T, e.g., for *ARID1B*, *MAP3K1*, *TP53* (A & B, black stars with coloured outlines in blood graphs).

The B panel was evaluated in 114 FFPE and in 89 blood samples, and the T panel in 69 and 44 samples, respectively (**Fig 1B**). Amplicon read ratios in blood and tumor samples are shown in **Tables B and D in S1 File**. For the B-panel, mean, median, ±SD values of amplicon read ratios in blood samples were 0.27, 0.26, ±0.12 and in FFPE 0.27, 0.26, ±0.19. Similar values were obtained for the T panel, 0.35, 0.34, ±0.14 in blood and 0.35, 0.32, ±0.18 in FFPE samples. Paired comparisons of blood—tumor differences in amplicon read ratios were not statistically significant (B panel: 95% CI: (0.00 to 0.04), paired t-test p = 0.0765; T panel: 95% CI: (-0.01 to 0.03), paired t-test p = 0.3029). Similarly, amplicon read ratios strongly correlated between the two different sample types with Spearman’s rho = 0.6055 (95% CI: 0.54–0.67) for the B-panel and 0.8212 (95% CI: 0.78–0.86) for the T-panel (both p’s <0.0001). In the same line, mapping all amplicon read ratios for all samples, both sample types and both panels, also revealed similar patterns of amplification efficiency per gene (**Fig 2**). As shown, however, read ratios for the same amplicons occasionally varied between panels in the blood sample series (germline), indicating different amplification efficiencies in different multiplexing environments; amplicons for genes with frequent gains or losses in breast cancer respectively yielded higher or lower read ratios in the corresponding tumor series as compared to germline; and, individual amplicons yielded unexpected high read ratios in tumors only, also indicating over-representation of the corresponding targets in the FFPE sequencing template.

**Fig 3:**
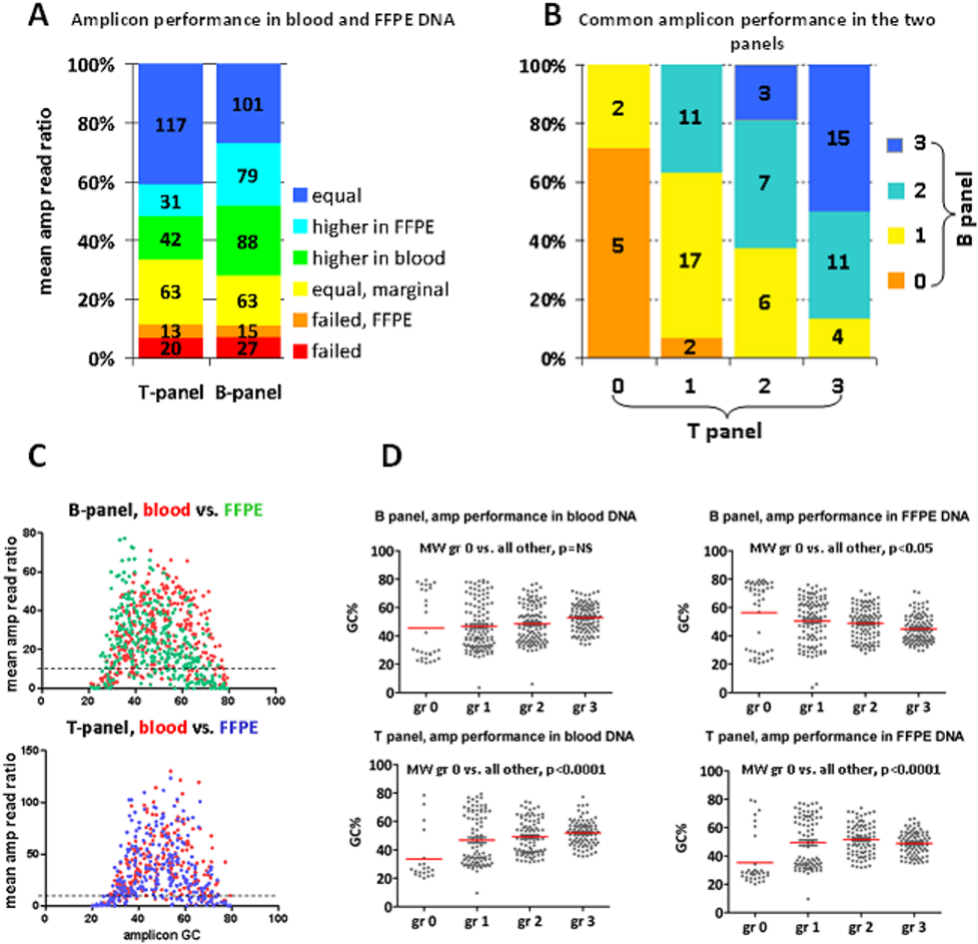
Performance of individual amplicons in blood and FFPE DNA. **A.** Amplicon performance grading did not significantly differ between the two panels. Columns: combined evaluation of each panel in blood and FFPE. Numbers within boxes: actual amplicon number per category, as indicated. **B.** Performance of the 83 common amplicons was significantly different in the two panels (p<0.0001). The most unstable amplicons were of performance grade 1 and 2. **C.** A non-linear distribution of mean read ratios according to amplicon GC content was observed. Read ratios of amplicons with >75% or <25% GC content were almost uniformly below the 10^th^ percentile cut off (dotted line in both graphs) (Kruskal-Wallis test p<0.0001 for each panel and sample group). **D.** Very high and very low GC content was significantly associated with failed amplicons (gr 0). This pattern was also present for the B-panel in blood samples, despite the absence of statistical significance. Except for these extreme cases, however, all other amplicon categories did not significantly differ with regards to GC%.

The ratio of eligible amplicons per sample with each panel was almost constant in the four sample groups; in the B panel series, the mean ratio (±SD) of eligible amplicons was 89.6% (±4.2) with min-max range at 79.4–96.8; corresponding values for the T panel were 84.7 (±10.3) with min-max at 50.7–95.8. Out of the 373 amplicons in the B-panel, 42 (13.1%) were classified as failed (grade 0) for FFPE and 27 (7.2%) for blood DNA, i.e., they had read counts of <125 in 90% of corresponding samples. Similarly, out of the 286 amplicons in the T-panel, 33 (11.5%) and 20 (7.0%) amplicons failed for FFPE and blood samples, respectively (**Fig 3A**). The above difference was not statistically significant between panels. As indicated in **Fig 2**, common and shifted amplicons (as per design criteria) performed significantly differently in both panels (Pearson’s p’s<0.0001), which was more prominent for failed and marginal (grade 0 and 1) amplicons (**Fig 3B**). Amplicon GC% was related in a non-linear fashion with amplicon read ratios for each panel and for each sample group; by using quartile cut offs (25%, 50% and 75%) for GC% categorization, very low and very high GC% was adversely associated with amplicon read ratios (**Fig 3C**). In line with this observation, amplicon performance (**Fig 3D**) was also significantly affected by extreme GC content. Failed amplicons had >75% or <25% GC; the latter was more prominent in the T-panel, almost non-overlapping with the rest of amplicon performance grades.

### Genotype comparisons with the two panels

In total, 2121 variants were considered eligible for the 89 blood-FFPE pairs with the B-panel, and 411 variants for the 44 blood-FFPE pairs with the T-panel. For the B-panel, 801 variants (37.8%) were found in both sample types (common variants), 141 (6.6%) in blood only, and 1179 (55.6%) in tumor samples only (**Fig D in S4 File**). For the T-panel, 141 (34.9%) common, 43 (10.4%) blood-only and 227 (54.7%) tumor-only variants were identified (**Fig E in S4 File**). At the applied read depth, tumor-only variants would correspond to somatic changes and were not considered for genotype comparisons in blood/tumor series. In comparison, because germline variants present in blood are expected to be present in tumour samples which also contained non-cancerous elements, blood-only variants were considered discordant. Blood-only variants were identified at germline zygosity frequencies (42–56% and 98–100%); none of these had known implications on protein function, almost all were annotated with a dbSNP ID, and for the B panel at least they were identified by best performing amplicons (grade 3 in blood and tumour). Their absence in tumor tissue DNA might correspond to loss-of-heterozygosity in breast tissue and the tumor itself. With respect to common blood/tumor changes, variant frequencies (VF) in blood were generally not preserved in tumors with the B panel (**Fig D in S4 File**), although they significantly correlated with the T panel (**Fig E in S4 File**). No technical reasons seemed to be implicated in the observed differences between blood and tumour genotypes.

**Fig 4:**
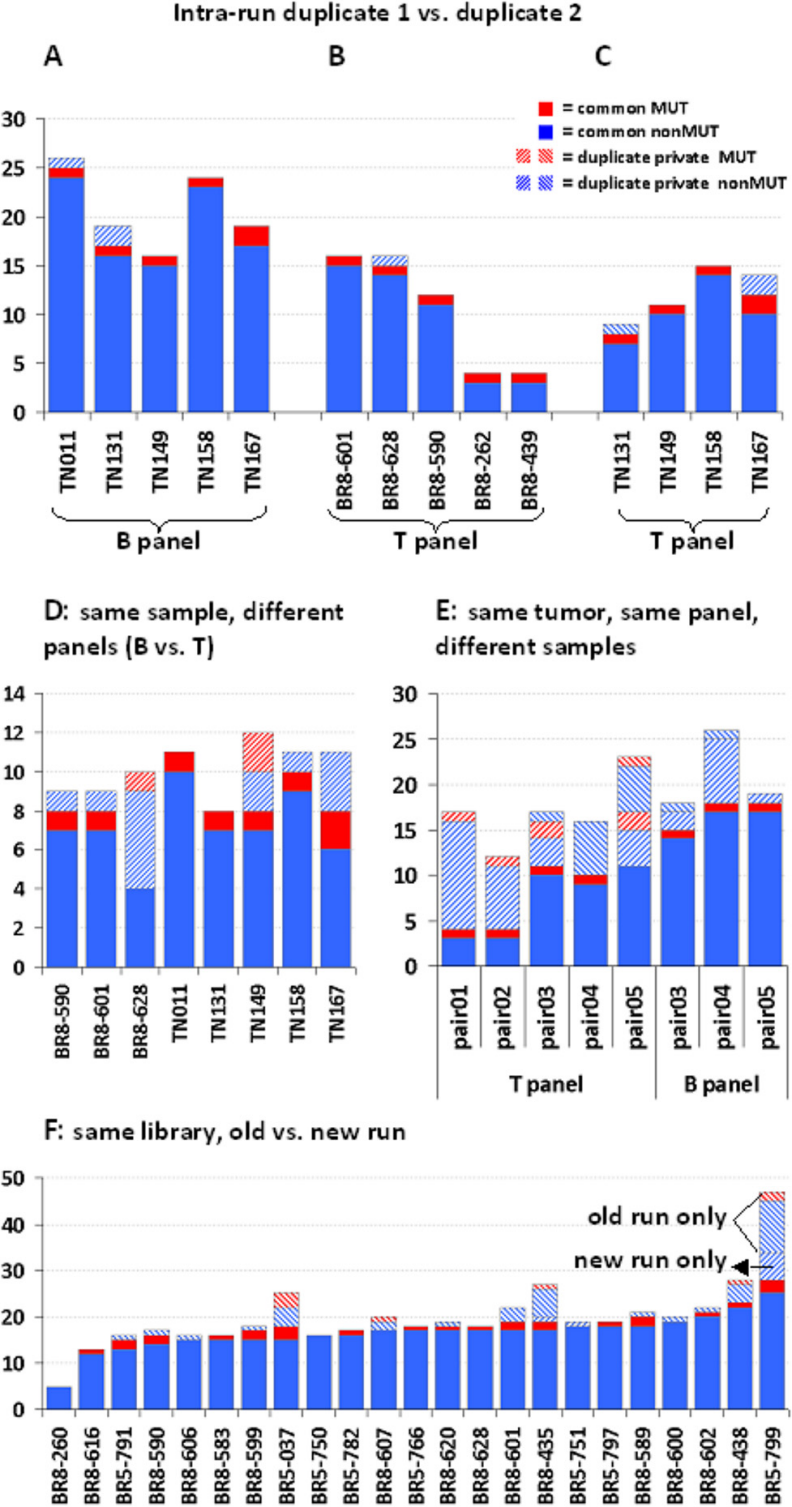
Variant concordance in FFPE samples. For all graphs, solid color boxes represent concordant, whereas striped boxes represent discordant variants. Red color stands for MUT variants (amino acid changing, excluding *TP53* p.P72R) and blue for nonMUT (non amino acid changing, coding and non-coding). Opposite directions of stripes stand for each paired sample. **A:** Five TN samples with the B panel. **B:** Five BR samples with the T panel. **C:** Four TN samples with the T panel (out of five on trial, one was ineligible). **D:** Panel comparison for the same sample. Only common and shifted amplicons in the two panels were evaluated. **E:** Different samples from the same tumor revealed distinct genotypes when tested with the same panel. **F:** Comparison between genotypes from subsequent runs performed 6 months apart for BR samples with the B panel. Old run only, new run only: patterns apply to all columns in this graph.

Reproducibility was evaluated in 152 datasets obtained from 25 TN and 25 BR samples that were tested with both panels in various combinations (**Table 1, Fig 4**). Run metrics and overall performance of these samples are presented in **Tables A—G in S3 File**. Genotypes were compared in the following groups: FFPE library duplicates in the same run, for the evaluation of intra-run reproducibility (n = 9 for the T and 5 for the B panel); application of the two panels on the same DNA sample, for the evaluation of primer/amplicon performance in different multiplexing environments (n = 8); comparison of the same panel on different DNA from the same tumor for considering technical issues when evaluating tumor heterogeneity (n = 8); and, inter-run comparison of genotypes for the same libraries (n_B-panel_ = 23 and n_T-panel_ = 23).

**Table 1:** Evaluation of variant calls with the B- and T- panel in paired FFPE sample and data groups.

	intra-run, duplicates, same library						inter-run, same library	
	total	*group 1*	*group 2*	*group 3*	group 4	group 5	group 6	group 7
**sample pairs, N**	14	*5*	*5*	*4*	8	8	23	23
**sample series**	BR & TN	*BR*	*TN*	*TN*	BR & TN	BR & TN	BR	TN
**panels**	B & T	*T*	*B*	*T*	B & T	B & T	B	T
**variant replicates**	208	*104*	*54*	*50*	86	148	459	366
**discordant variants**	7	*3*	*1*	*3*	20	57	54	130
**mean concordance**	97.24	*97*.*13*	*99*.*63*	*94*.*4*	81.01	59.9	91.59	65.45
**(±SD)**	4.66	*4*.*59*	*0*.*83*	*6*.*7*	20.35	24.03	11.01	15.69
**95% CI**	94.80–99.68	*93*.*11–101*.*15*	*98*.*90–100*.*36*	*87*.*83–100*.*97*	66.91–95.11	43.25–76.55	87.09–96.08	59.04–71.86
**min—max** ^	86.66–100	*89*.*47–100*	*98*.*15–100*	*86*.*7–100*	40–100	23.53–94.74	59.60–100	38.50–100
**p-value** *	<0.001	* *	* *	* *	0.004	0.282	<0.001	<0.001

N: number; group 4: same DNA sample, different panels; group 5: same panel & tumor, different DNA samples; SD: standard deviation; CI: confidence interval

^: min—max concordance in individual sample pairs in each group

*: one sample t-test.

In total, 1267 eligible calls in coding and non-coding regions of the targeted genes were compared (**Table 1**). Out of these, 999 (78.8%) were observed for both samples of each trial pair and were considered as concordant. The remaining 268 variants (21.2%) were observed in either sample of each pair and were considered as discordant. Among all variants, 111 were amino acid changing (8.7%), 35 of which were discordant (13.0% among all discordant and 31.5% among amino acid changing variants). For genotype comparisons, all variants resulting in amino acid change (MUT), as well as those producing silent changes in coding regions or nucleotide changes in non-coding regions (nonMUT) were evaluated. TP53 p.P72R polymorphism (rs1042522) was considered as nonMUT. The highest concordance was observed for intra-run duplicates with the B and T panels (**Table 1**); identical genotypes for coding and non-coding variants were obtained for 9 out of 14 compared sample pairs (**Fig 4A, 4B and 4C**), with only nonMUT changes in either duplicate when present. In libraries constructed with the B or T panel from the same DNA samples, the concordance of variants detectable with identical amplicons or with amplicons covering common targets in both panels was modest with 2 identical genotypes among 8 samples (**Fig 4D**). Testing different DNA samples from the same tumor with the same panel resulted in different genotypes among all paired groups (**Fig 4E**). Finally, inter-run comparison of BR samples with the B panel revealed 8/23 identical genotypes; in the remaining cases unpaired variants mostly corresponded to the old run (**Fig 4F**) indicating that library stability declines over time. By contrast, in the corresponding setting with TN samples and the T panel, unpaired variants were mostly from the recent run (**Fig F in S4 File**). This series may serve as an example of how run quality metrics may affect variant calling and overall sample performance (**Table G in S3 File)**; reading efficiency in the new as compared to the old run was more than double, while sample performance with respect to uniformity and number of eligible amplicons was accordingly higher.

### Impact of amplicon performance, coverage and variant frequency (VF) on genotype reproducibility

In order to further understand the observed differences in paired genotypes, we compared the obtained variants against sequencing system and bioinformatics parameters. Insertions/deletions (INDELs) were less common (98/1267 [7.7%]) than single nucleotide variants (SNVs) (1158/1267 [91.4%]) but showed a trend for higher incidence among discordant variants (Pearson’s p = 0.063); in this context, discordance concerned polyC stretches, as widely discussed for this technology. Multiple nucleotide variants were rare. Discordant calls were more frequent among G>C transversions, while the rate of discordant, repeatedly called amino acid changes for some genes, e.g., *GATA3*, *PIK3CA*, was particularly high (**Fig G** and **Fig H in S4F File**). With respect to amplicon performance, discordant variant rates for grade 0 amplicons (57.6%) were significantly higher in comparison to all other grades, for example to grade 3 amplicons (7.7%) (p<0.0001) (**Fig I in S4 File**).

**Fig 5:**
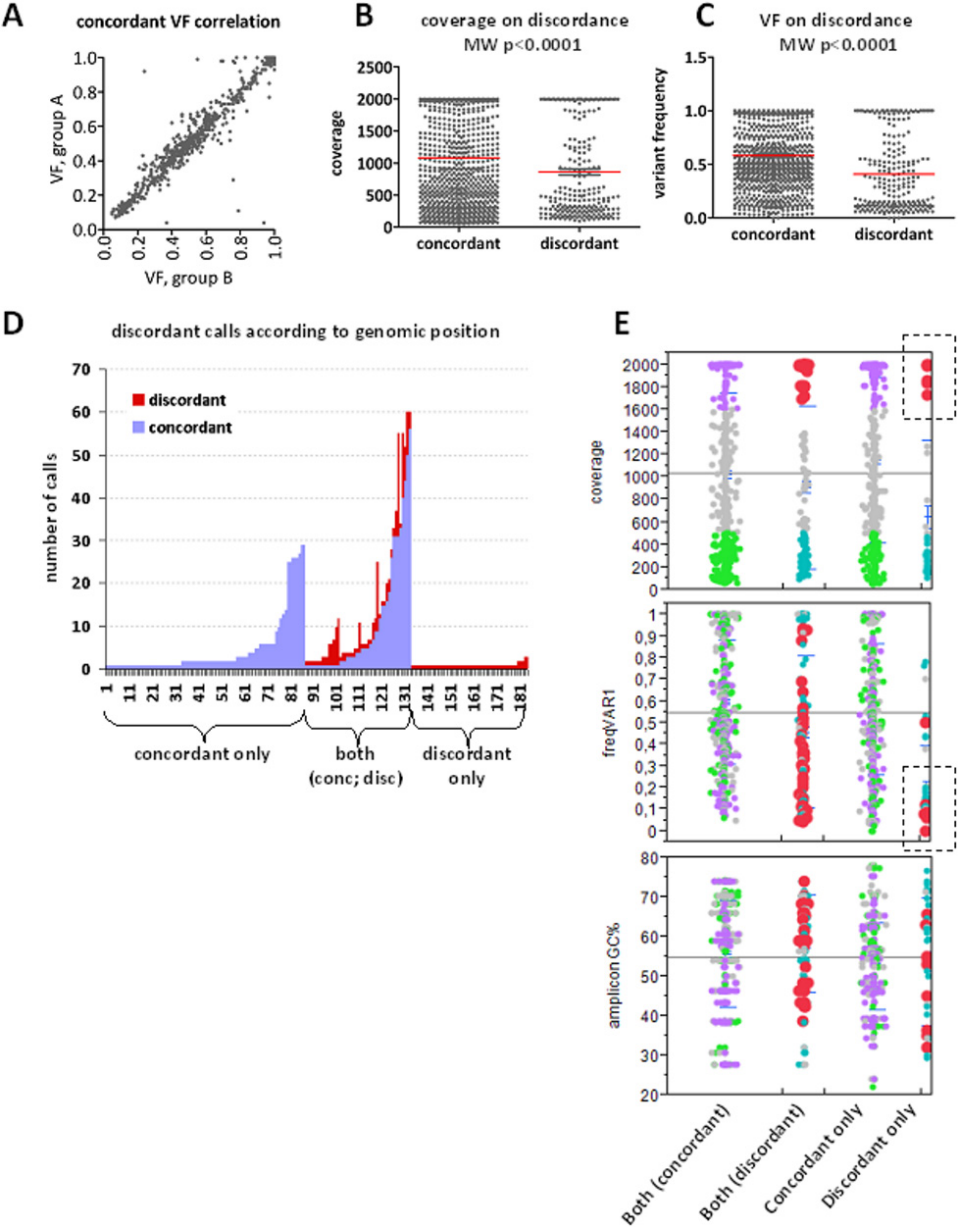
Effect of coverage and frequency on variant concordance with custom panels on FFPE samples. **A:** Variant frequency (VF) was highly preserved in replicate measurements from the same samples. **B and C:** Concordant variants had statistically significant higher coverage and frequency as compared to discordant ones (red lines: mean values). However, both high and low coverage and frequency were observed for discordant variants and followed the same bimodal pattern in these categories. **D.** Out of the 181 different positions accounting for the 1267 variants under study, calls were constantly concordant in 83 (45.8%), partially concordant in 26.0%, and constantly discordant in 28.2%. **E.** Distribution of VF and amplicon GC% according to high and low coverage for the categories indicated on the bottom of the graphs. Red and lilac dots: high coverage in discordant and concordant variants, respectively; turquoise and green dots: low coverage, respectively. Among discordant only variants, those of high coverage were almost exclusively of low VF (boxed).

Variant frequency (VF) was retained in paired genotypes among concordant variants, especially in datasets from the same samples irrespectively of the panel used (**Fig 5A**), with Spearman’s rho 0.959 (95% CI 0.953–0.964; p<0.0001).

Position coverage (**Fig 5B**) and variant frequency (VF) (**Fig 5C**) was significantly lower in discordant as compared to concordant variants (mean [±SD] 0.41 [±0.34] vs. 0.58 [±0.30], median 0.30 vs. 0.53, respectively, for VF). However, for both coverage and VF a bimodal pattern was observed with respect to discordance. In this context, except for the expected low coverage and low frequency, discordant variants of high coverage and high VF were also observed. The 1267 variants were identified at 181 different positions (genomic coordinates) corresponding to 136 amplicons; exclusively discordant calls were observed at 51 positions, while at 47 additional ones both concordant and discordant variants were identified (**Fig 5D**). High coverage strikingly corresponded to low VF variants for positions with exclusively discordant calls (**Fig 5E**).

The above associations are reflected in paired genotypes for amino acid changing variants, as presented in **Table 2** for 5 paired DNA samples. The listed variants are COSMIC registered amino acid changes, all considered as protein function affecting mutations except for *TP53* p.P72R; the latter is shown since it is the most common *TP53* variant. Revision of raw data for variant coverage and amplicon reads was performed in order to address discordant calls. With respect to **Fig 4D**, discordant mutation calls with the B and T panels corresponded to *PTEN* p.Asp24Gly for sample BR8-628 and to *PIK3CA* p.His1047Arg and *TP53* p.Arg209Lys for sample TN149. In all these cases, sufficient amplicon reads were obtained. With respect to **Fig 4E**, it is shown that (a) low VF variants detected at high coverage with one panel could not be detected with the second panel, e.g., *PTEN* and *PIK3CA* mutations were present in duplicates with the same panel; (b) high VF variants were detected at low coverage with both panels, e.g., *TP53* p.Pro72Arg for TN011 (T panel) and BR8-601 (B panel); (c) observing amplicon reads was necessary for the evaluation of genetic heterogeneity in the same tumor in order not to misinterpret failed amplicons, as in TN167 and BR8-439 where unpredictability of sample vs. panel behaviour should be implicated; and (d) tumors appeared as heterogeneous or not for the genes tested according to the panel used, as indicated.

**Table 2:** Comparison of genotypes for amino acid changing variants in paired panel and sample series.

sample ID (1)	TCC	panel	variant coverage	VF	gene	protein	sample ID (2)	TCC	panel	variant coverage	VF	gene	protein	tumor heterogeneity
BR8-262	70	T	136	0,28	*TP53*	p.Arg273Cys	TN158	50	T	1372	0,29	*TP53*	p.Arg273Cys	potentially
BR8-262		T	*amplicon failure (16 reads)*				TN158		T	1192	0,92	*TP53*	p.Pro72Arg	heterogeneous
BR8-262		T	*AR 416*, *no variant*				TN158		T	794	0,38	*TP53*	p.Arg175Gly*	(TP53)
BR8-439	55	T	728	0,14	*PIK3CA*	p.His1047Arg	TN167	60	T	1996	0,47	*PIK3CA*	p.His1047Arg	inconclusive
BR8-439		T	*amplicon failure (16 reads)*				TN167		T	796	0,26	*TP53*	p.Pro72Arg	
BR8-439		T	*amplicon failure (45 reads)*				TN167		T	1239	0,46	*TP53*	p.Tyr163Cys	
BR8-590	65	T	1992	0,31	*TP53*	p.Tyr107*	TN149	85	T	1996	0,24	*TP53*	p.Tyr107*	heterogeneous
BR8-590		T	*AR 659*, *no variant*				TN149		T	1986	0,29	*TP53*	p.Pro72Arg	(TP53, PIK3CA)
BR8-590		T	*AR 1494*, *no variant*				TN149		T	2000	0,12	*PIK3CA*	p.His1047Arg	
BR8-590		T	*AR 666*, *no variant*				TN149		T	1966	0,17	*TP53*	p.Arg209Lys	
BR8-590		**B**	544	0,36	*TP53*	p.Tyr107*	TN149		**B**	1996	0,75	*TP53*	p.Tyr107*	no heterogeneity
BR8-590		**B**	*AR 408*, *no variant*				TN149		**B**	*AR 798*, *no variant*		*TP53*	p.Pro72Arg	(discordant B and T
BR8-590		**B**	*AR 1754*, *no variant*				TN149		**B**	*AR >4000*, *no variant*		*PIK3CA*	p.His1047Arg	panel results for
BR8-590		**B**	*AR 259*, *no variant*				TN149		**B**	*AR 940*, *no variant*		*TP53*	p.Arg209Lys	TN149)
BR8-601	60	T	222	0,84	*TP53*	p.Pro72Arg	TN011	90	T	65**	0,92	*TP53*	p.Pro72Arg	no heterogeneity
BR8-601		T	1994	0,26	*PIK3CA*	p.His1047Arg	TN011		T	1550	0,47	*PIK3CA*	p.His1047Arg	(TP53, PIK3CA)
BR8-601		**B**	364	0,23	*PIK3CA*	p.His1047Arg	TN011		**B**	1994	0,49	*PIK3CA*	p.His1047Arg	no heterogeneity
BR8-601		**B**	72**	0,72	*TP53*	p.Pro72Arg	TN011		**B**	153	0,76	*TP53*	p.Pro72Arg	(TP53, PIK3CA)
BR8-628	65	T	1999	0,15	*PTEN*	p.Asp24Gly	TN131	75	T	*AR 1697*, *no variant*				heterogeneous
BR8-628		T	455	0,44	*TP53*	p.Pro72Arg	TN131		T	1391	0,10	*TP53*	p.Pro72Arg	(PTEN, TP53)
BR8-628		T	*AR 1047*, *no variant*				TN131		T	1666	0,79	*TP53*	p.Thr155Pro	
BR8-628		**B**	*AR 1912*, *no variant*				TN131		**B**	*AR 892*, *no variant*				no heterogeneity
BR8-628		**B**	403	0,18	*TP53*	p.Pro72Arg	TN131		**B**	898	0,09	*TP53*	p.Pro72Arg	(discordant B and T
BR8-628		**B**	1101	0,67	*TP53*	p.Thr155Pro	TN131		**B**	1947	0,82	*TP53*	p.Thr155Pro	panel results for
														BR8-628)

TCC = tumor cell content (%); VF = variant frequency; AR = amplicon reads

*: variant observed with the old library with the same panel for the same DNA sample

**: low coverage / high frequency variants that were initially missed with the 125 coverage cut-off.

### FFPE Sanger sequencing validation of MPS variants

**Fig 6:**
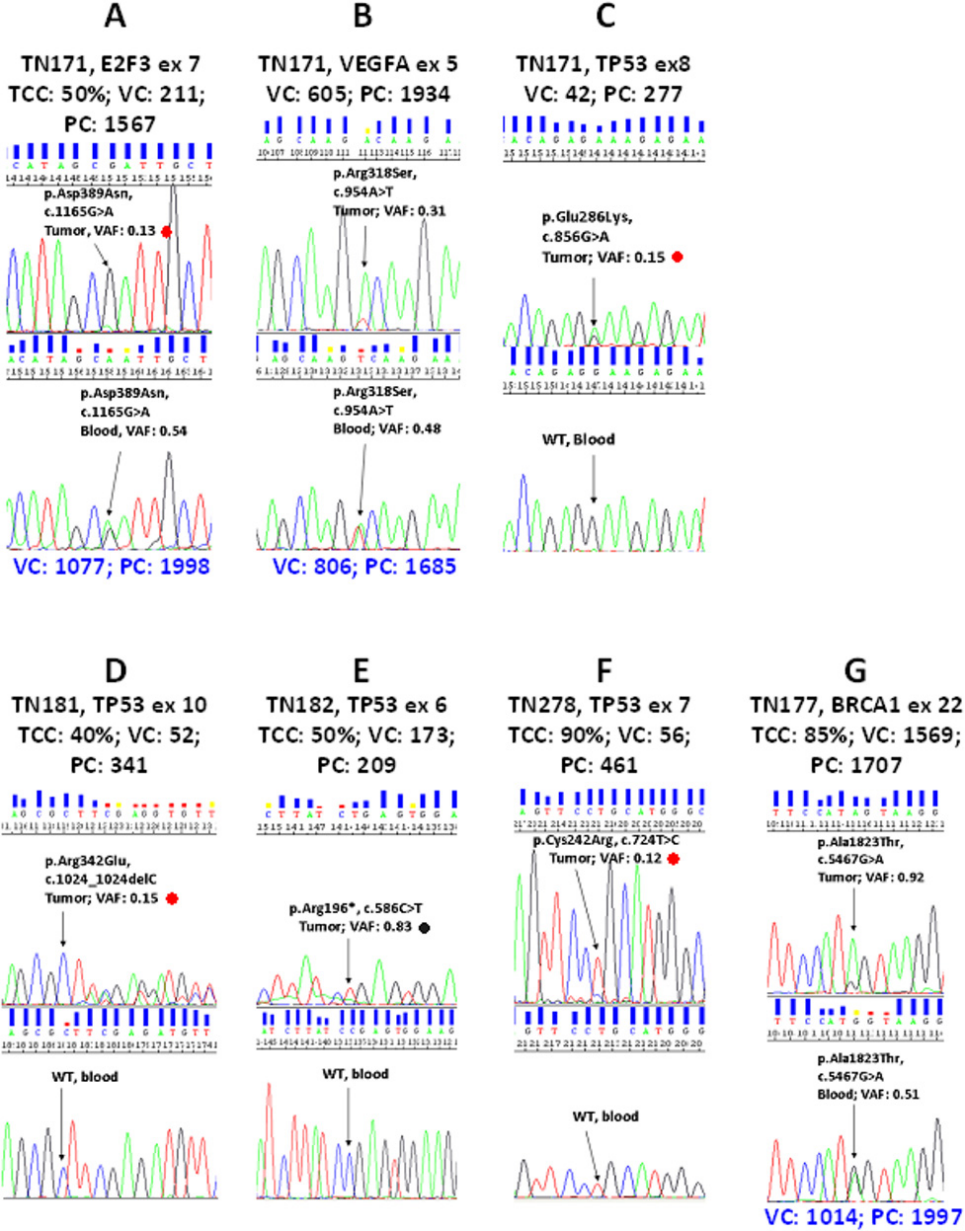
Sanger sequencing validation of MPS variants. Five cases with matched tumor—blood samples are shown. Annotations in black letters are given for tumors (FFPE); in blue letters for germline (blood). TCC: approximate tumor cell content in the DNA sample; VC and PC: variant and position coverage with MPS; VAF: Variant frequency (VC/PC). The same DNA samples were used per case for both methods. A—C: same case, DNA quality unfavourable, three variants in tumor, two in germline. D—F: individual cases with unfavourable (D and E) and favourable (F and G) DNA quality. Sanger sequencing peaks usually but not always corresponded to MPS VAF. In A, D and F, perhaps in B as well, variants would have been missed with Sanger sequencing only. Note target specific differences in position coverage, which derives from amplicon reads. Red dots: low VAF. Black dot: the expected wild type allele at 17% frequency, based on MPS VAF, was not observed with Sanger sequencing.

MPS variants for the genes tested were validated in all 50 cases (FFPE and blood DNA). It was possible to validate MPS variants with Sanger sequencing even in FFPE samples with unfavorable quality and TCC metrics (**Fig 6**). Among the 5 cases in this figure, FFPE DNA was of predicted good quality (qPCR average cycle threshold [CT] for wild-type alleles at 29; >2ng DNA/ul) in 2 only (**Fig 6F and 6G**), while quality metrics in the remaining 3 cases were suboptimal (qPCR CT ~32; 0.5–1ng DNA/ul). DNA quantity and quality did not necessarily determine target amplification efficiency, which was amplicon specific (compare **Fig 6A and 6B** vs **6C** in the same case; and, good quality DNA in **Fig 6F** vs **6G**), while variants were reproducible even if covered <50 times. For example, a tumor-specific *TP53* single nucleotide deletion identified at a frequency of 15% with MPS could be validated in the TN181 FFPE DNA sample containing 40% tumor cells (**Fig 6D**). Except for this variant, however, which causes a shifted electropherogram, low frequency variants such as in **Fig 6A, 6B and 6F** would have been difficult to interpret with Sanger sequencing only, since these were returned as very low peaks. Further, if comparing TCC%, variant frequency in the tumor, and variant presence in the germline, it seemed that the *E2F3* and *VEGFA* germline variants in **Fig 6A and 6B** were in fact not retained in the corresponding tumor. A detailed blood—tumor variant comparison and evaluation for biologic implications is beyond the scope of the present study.

## Discussion

In this study we evaluated the performance of two highly multiplexed custom panels for MPS on DNA from routinely processed FFPE DNA samples. Statistical comparisons of the obtained amplicon read, variant calling and variant coverage metrics with both custom panels were in line with previous reports all of which refer to the commercially available Ampliseq Cancer panels for mutation hotspots. Amplicon read efficiency in blood, i.e. good quality, and FFPE, i.e. poor quality DNA samples were comparable with both panels, as published for frozen—FFPE tissue comparisons [20, 38]. Genotyping for mutations in library duplicates in the same run was 100% concordant with both panels, as reported in [18]. Finally, considering panel overall performance, no significant difference was observed with either type of material, despite that the panels differed by ~90 amplicons (180 primers).

A positive finding from the present study with custom panels is that once detected, variants were called at the same frequency upon repeated testing of FFPE libraries with any panel. This supports the use of variant frequency as a metric for the evaluation of heterozygosity and for assessing allelic imbalance among FFPE samples, as already applied [39]. Reproducible results for germline SNPs were also obtained with blood—tumor sample pairs, further supporting the reliability of MPS results. The fact that germline variant frequencies were not retained in matched FFPE samples may be related to somatic changes in tumor DNA and not to technical errors, as exemplified in the *E2F3* and *VEGFA* germline variants that were validated with Sanger sequencing. Also, it may be an effect of the type of tumors analyzed, since we observed different rates of variant frequency similarity in TNBC series as compared to the series including all breast cancer subtypes. This is the objective of an ongoing investigation in larger series by our group. The findings presented here, however, do suggest that unusual variants found in tumor tissue samples, which are always contaminated with non-tumor DNA, may not necessarily be somatic, and that assessing variant frequency with respect to TCC for the identification of intratumoral mutation frequency without taking into account germline data, may be misleading. The same findings on these randomly selected variants from our MPS results also point out the importance of custom panel-based research, since *E2F3* and *VEGFA* are practically not addressed for mutations, especially in the germline context.

Issues highlighted in this study with respect to FFPE MPS data evaluation and interpretation are (a) the need for having in hand the entire sequencing status while evaluating variants, i.e., whether the target sequences have been read in the first place; and (b) the need to individualize criteria for accepting variants due to the unpredictable behavior of FFPE DNA. Starting with the first point, libraries generated from the same DNA sample amplified with different panels occasionally yielded different genotypes for positions that were targeted with common or slightly shifted amplicons. One explanation for this observation is that, as shown, the performance of amplicons with the same or slightly shifted primers significantly varied in the different multiplexing environments of the two panels. This indicates multiplexing selectivity and is in line with primers behaving uniquely upon multiplexing as has recently been shown upon attempts to resolve discordant findings with the Ampliseq Cancer panel itself [10] and with certain amplicons continuously producing wrong reads [18]. Currently, the only way to check library templates is fluorometry, i.e., general nucleic acid quantification, which is far from sufficient for testing multiplexing efficiency. In this context, cost-efficient technological achievements allowing for tracking amplified amplicons within a library would be more than welcome and would help avoiding the sequencing procedure for libraries with poor amplicon representation.

Libraries with our custom panels were created after initial amplification of DNA with 286–370 primers in each pool. Given this complexity, it is expected that not all amplicons will amplify in the same way and that some may not amplify at all in every DNA sample, in line with observations upon validation trials for diagnostic panels [11, 18]. Here, we classified amplicons according to their cumulative performance in 90% of the samples, which was helpful for interpreting discordant results. However, our stringent classification, similarly to the proposed 20% cut-off for accepting specific variants [6, 11] is retrospective and can certainly not be applied in any algorithm for the prospective validation of genotypes, since failed amplicons occasionally produced correct results and *vice versa*. We have further shown that amplicon performance depended on their GC content in a binomial mode, as expected [40]. As also stated by Golan & Rosset in [40], the impact of GC content on amplicon performance and amplicon performance *per se* are not constant across experiments. In addition, as with Sanger sequencing, MPS amplicon performance did not necessarily depend on input DNA quality/quantity metrics in our FFPE samples but on the condition of the targeted sequence in the given sample. Each FFPE sample should a priori be considered as unique. If we accept this condition, targeted sequences, whether one at a time, as with qPCR or with Sanger sequencing, which is not free of wrong calls [18, 41], or several hundreds, as with MPS, should be evaluated for efficient amplification individually in each sample in order to judge for specificity and sensitivity of called variants. Therefore, it would be very useful to automatically obtain raw data on the performance of all amplicons in combination with variant calls, a software feature which is not yet available.

Our data on the positive association of high coverage and high variant frequency on variant reproducibility are in line with previous reports with Ion Torrent [12] or with Illumina sequencing [42] on DNA from various sources. As shown, however, high coverage may not be adequate as a single metric for accepting eligible variants. Variants from positions covered close to, or higher than 2000 times were not constantly replicated in repeated trials of the same library, especially if they were of low frequency. In such cases, 15% frequency corresponded to >300 qualified reads for the particular variant. Thus, reproducibility of high coverage / low frequency variants appears unpredictable, indicating that deeper sequencing for the identification of more variants may yield non-replicable results. These results are concordant with increasing genotype error rates when increasing reading depth [43] and with low variant frequencies returning false positive results for certain genes [12], although lack of orthogonal variant validation may not necessarily mean error. With respect to low coverage and frequency, variants read 50 times from positions read less than 100 times were reproducible, other than recently stated for formalin induced sequence artefacts [44], while variants read 40 times at 10% frequency were orthogonally validated. Such variants would go unnoticed if strictly adhering to cut-offs for amplicon and position reads at 125, for variant coverage at 50, and for variant frequency at 20%, as initially set here. Thus, it appears that for FFPE DNA, amplicon reads, position and variant coverage, and variant frequency should be assessed as a combined parameter for accepting variants for further evaluation.

Related to and based on all the above, caution is needed in the interpretation of genetic tumor heterogeneity which is currently one of most frequently addressed research topics. Tumours are reported as heterogeneous with holistic approaches [45], while recently intra tumour heterogeneity has been interpreted with the AmpliSeq Cancer panel [20] and with a custom panel [22]. Genotype and tumour heterogeneity comparisons with different highly-multiplexed panels have not yet been reported, to our knowledge. As shown here for known mutations in the genes targeted, tumors called heterogeneous with one panel would have been called homogeneous if tested with the second panel and *vice versa*. Evaluation of amplicon reads and revisiting variants with low coverage eliminated false negative results and false positive heterogeneity. The observed discrepant genotypes with the two panels, mostly but not entirely, seemed to be due to different primer efficiencies in different multiplexing environments especially for low variant frequencies, as previously reported [10]. Cytosine deamination has been blamed for the increased incidence and lack of reproducibility of C:G>T:A mutations [46, 47]. However, discrepant genotypes in our case did not only correspond to transition changes, while UDG pretreatment of DNA may not always be adequate for reducing artifacts [47]. The herein revealed panel specificity of the obtained genotypes is an emerging issue to be considered when interpreting results with targeted MPS.

In conclusion, MPS with highly-multiplexed custom panels is feasible with semiconductor sequencing for large scale analyses with FFPE DNA and may reliably yield genomic aberrations not previously described. It should be kept in mind though that FFPE DNA from tumor tissues is a fundamentally different context as compared to DNA from peripheral blood for the evaluation of germline variants. With respect to tumor tissue particularities, in the absence of person-private non-tumor DNA data, interpreting variants as tumor-specific may be misleading. As with any molecular method that has been developed for the analysis of intact DNA and extended to fragmented FFPE templates, including the Sanger method for sequencing one target, it is of utmost importance to know whether and how the hundreds of targets in a custom panel have been read for each sample in the first place; combining this information with variant coverage and frequency would help assessing MPS results on a more solid ground.

## Supporting Information

S1 File Panels. Each panel is presented in detail in a separate xls sheet (Tables A and C); for each panel, all amplicon read ratios are shown in separate sheets (Tables B and D).
(XLS)

S2 File Patient and tumor characteristics. Contains Table A: BR (all breast subtypes); Table B: TN (triple negative breast cancer); and, Table C: sample pairs from the same tumor are presented in different xls sheets.
(XLS)

S3 File FFPE run and sample metrics. Includes Tables A—G with run metrics for FFPE samples in different xls sheets.
(XLS)

S4 File Supplemental methods and figures. Includes dd-sequencing protocol details and Figs A—I.
(DOC)
