# Supplementary material for: Evaluation of Two Highly-Multiplexed Custom Panels for Massively Parallel Semiconductor Sequencing on Paraffin DNA
Source: PLoS One. 2015 Jun 3;10(6):e0128818. doi: 10.1371/journal.pone.0128818 (PMC4454570; doi:10.1371/journal.pone.0128818)
Supplement: S4 File — Includes dd-sequencing protocol details and Figs A—I. (DOC) [file pone.0128818.s004.doc]

**Evaluation of two highly-multiplexed custom panels for massively parallel semiconductor sequencing on paraffin DNA**

*Kotoula V, et al*

**S4 File: Supplemental Methods and Figures**

**Figure A: Total amplicon coverage in all series examined with the two panels**


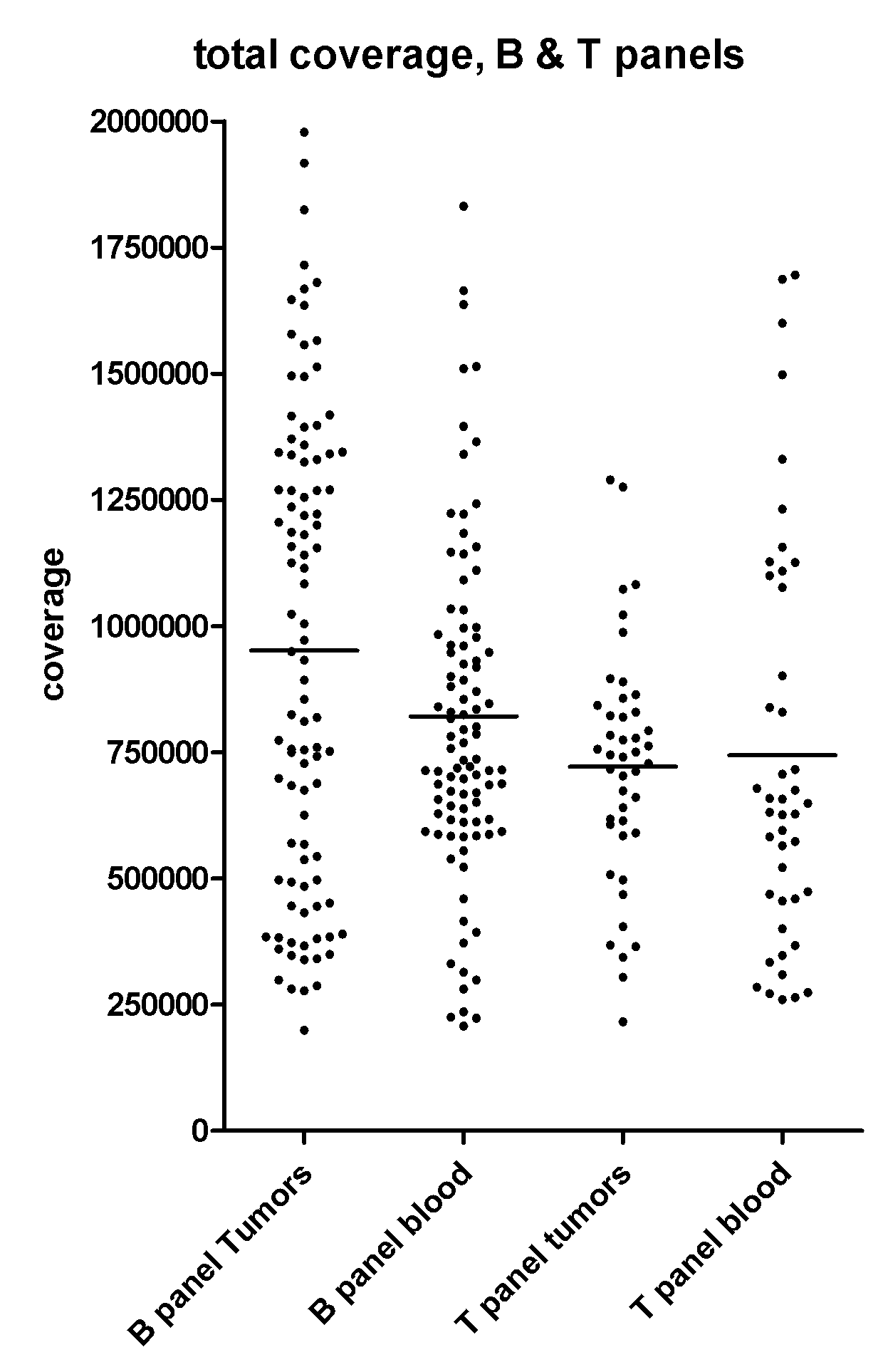


All samples were run at the same multiplexing (96 samples per PI chip). Graph shows total mapped reads, i.e., total amplicon coverage per sample. Five samples with the B panel with coverage >3000000 are not shown. Values were generally lower for the smaller T as compared to the larger B panel.

**Supplemental Methods: Confirmatory Sanger sequencing**

Sanger sequencing was performed for nested PCR products coupled with forward and reverse M13 primers at the 5’ end. Nested PCR primers, their coordinates and sequenced target lengths are shown in the Table below. PCR conditions were initial denaturation at 95°C for 10’, then 18 or 28 cycles (1^st^ or 2^nd^ PCR respectively) of denaturation at 95°C for 30’’, annealing at 60°C for 30’’, and primer extension at 72°C for 1’, and a final extension step at 72°C for 10’ for the nested reaction. Sense and antisense sequencing was performed in a 10ul reaction with the Big Dye Teminator kit v.1.1 (Applied Biosystems, Foster City, USA). Sequences were visualized upon capillary electrophoresis in an ABI3130XL genetic analyzer, and were initially called with the Sequencing Analysis software v.5.2.

**Primers used for Sanger sequencing in this study**

**Figure B: Examples of *PIK3CA* mutations detected using the Ion Torrent platforms and confirmed by Sanger sequencing.**

**
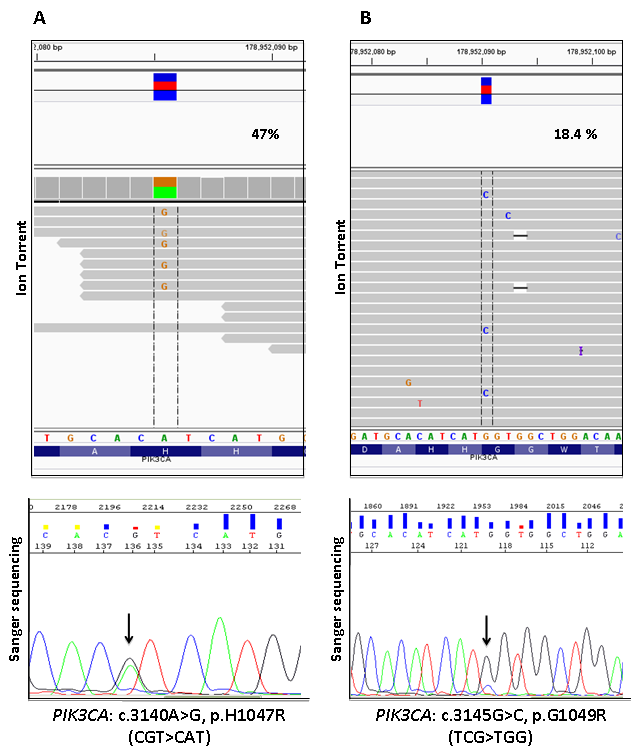
**

**A**. A substitution mutation (c.3140A>G, p.H1047R) which resulted from a CGT>CAT (histidine to arginine) change at codon 1047. **B**. A substitution mutation (c.3145G>C, p.G1049R) which resulted from a TCG>TGG (glycine to arginine) change at codon 1049. The mutations were evident in the forward strand in both cases and were confirmed by Sanger sequencing (arrows).

**Figure C: Examples of *TP53* mutations detected using the Ion Torrent platforms and confirmed by Sanger sequencing.**

**
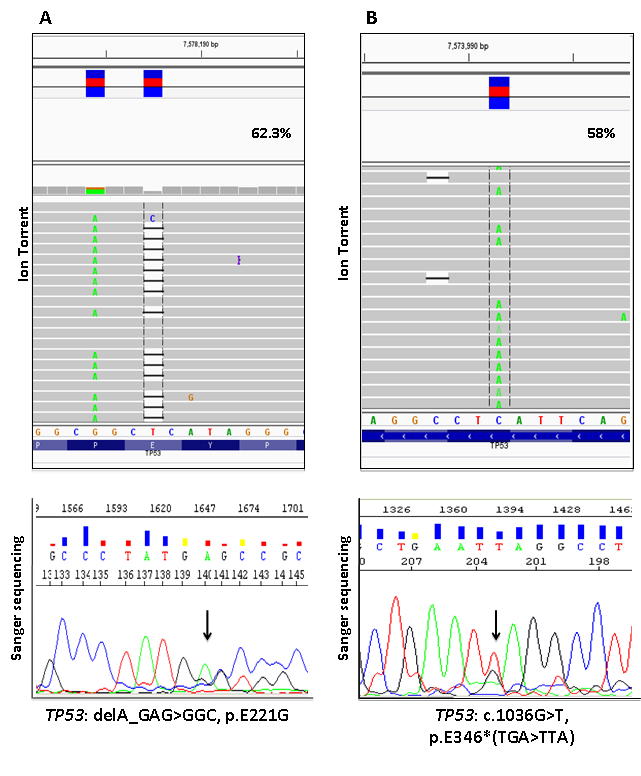
**

**A.** A deletion mutation (delA_GAG>GGC, p.E221G) evident in the reverse strand in the IGV and detected by Sanger sequencing (arrow). **B**. A truncating mutation (c.1036G>T, p.E346*) which resulted from a TGA>TTA (glutamic acid to stop codon) change at codon 346. The mutation was evident in the reverse strand in the IGV and detected by Sanger sequencing (arrow).

**Figure D: Variant calling in 89 paired blood – tumor BR samples with the B panel**

**
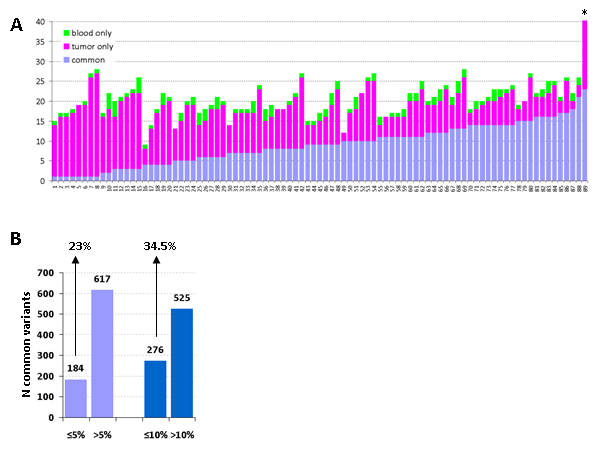
**

**A.** Considering blood variants as germline, blood-only variants (green) were considered discordant since they were expected to be present in tumor samples, which contained DNA from non-cancerous elements as well. In 85/89 cases, 1 – 3 blood-only variants were observed, irrespectively of the total amount of variants per case (*). Overall, concordance between germline and tumor excluding somatic variants was high (mean [±SD] 82.7 [±4.74], 95% CI 91.72 – 93.69, min-max 50 – 100%). Blood-only variants with the B-panel corresponded to SNPs with a dbsnp ID, some of which were repeatedly present. AMPL6083329467 identified rs34841024 and rs3769818, two A/G transitions in CASP8 intron 9 with equal minor allele frequency (MAF) for A=0.308/671, which were present in blood samples from 11 and 79 patients, respectively, but not in matched tumors; AMPL4050069285 identified rs80075693, again a C/T transition in TAOK1 intron 19 (MAF unknown) that was present only in blood DNA in 23 patients; rs10069690 and rs2242652, C/T (MAF T=0.318/693) and G/A (A=0.177/385) transitions in TERT intron 4 that were identified by AMPL3501685042 and AMPL3503455543, respectively, were found only in the blood from 6 patients. All above amplicons yielding blood-only variants performed equally well with blood and FFPE DNA (performance grade 3).

**B.** Similarity of variant frequency (VF) in blood and tumor DNA was tested for common variants as the % difference between VF_tumor_ – VF_blood_ with cut-offs arbitrarily set at 5% and 10%. Among 801 common variants in blood and tumor, VF was not preserved in the majority of the cases, while no correlation was observed in VF values between the two sample series (Spearman’s rho -0.057; 95% CI: -0.127 to 0.013). Amplicon GC%, amplicon performance grades or difference in amplicon performance between blood and FFPE samples were not associated with common blood-tumor VF status. Of note, all breast cancer subtypes were represented in the BR sample series, whereby only 7 were TNBC.

**Figure E: Variant calling in 44 paired blood – tumor TN samples with the T panel**


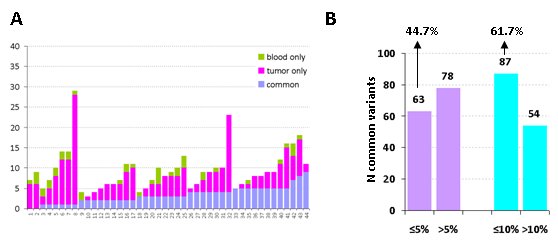


**A.** Similarly to findings with the B panel, 1 – 3 blood-only variants were observed in 24 out of these 44 TNBC cases with the T panel. Blood vs. tumor germline concordance was higher but not significantly different in this series as compared to the BR series (mean [±SD] 89.12 [±12.97], 95% CI 85.29 – 92.95; min – max: 93 – 100). With the T panel, 27 out of 43 blood-only variants were SNPs with a dbSNP ID, whereby the known polymorphism TP53 p.P72R (rs1042522, a G/C transversion) was noticed in 7 cases. With respect to blood-only variant genotypes, 25 out of 43 (58%) T-panel variants were C/T (G/A) transitions; this rate was significantly lower as compared to >90% transitions for blood-only variants with the B-panel (p=0.003).

**B.** Among 141 common variants in blood and tumor, variant frequency (VF) was not preserved in about half of the cases, while significant correlation was observed in VF values between the two sample series (Spearman’s rho 0.676; 95% CI: 0.574 to 0.756). As with the B panel, common blood-tumor VF status was not related to technical parameters. The different composition of the series tested with the B- and T-panel in breast cancer subtypes may have contributed to the observed difference in blood-tumor VF.

**Figure F: Comparison between genotypes from subsequent runs performed months apart for TN samples with the T panel.**

**
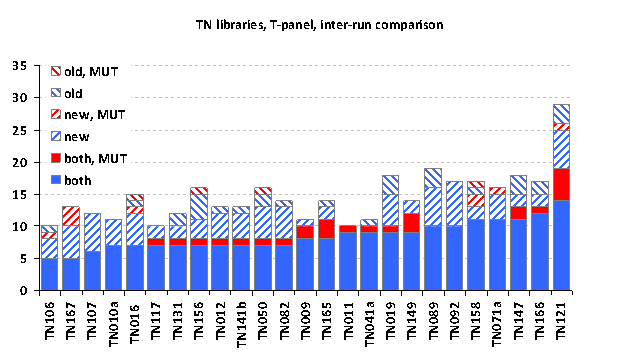
**

The time interval for this inter-run trial with the same libraries was 12 months. Run metrics are shown in File S3, TABLE S7.

**Figure G: Discordant variants according to nucleotide change**


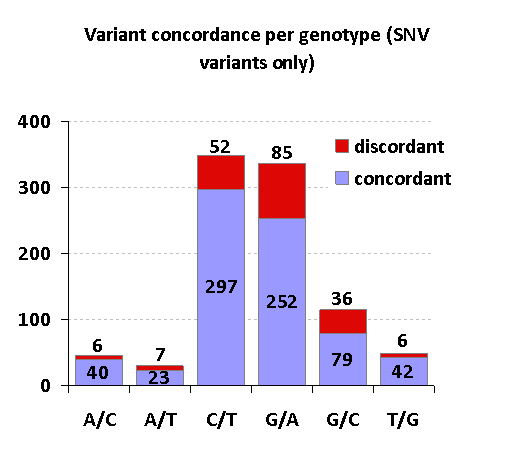


**variants per genotype (SNV only)**

G>C transversions were the most unreliable genotype, since 32.2% of such cases were discordant, although this change was only 3^rd^ in order among single nucleotide variants. This mostly reflected the high discordance observed for the commonly observed TP53 p.P72R G>C transversion, for which 24/55 (43.6%) of the calls were not replicated with either panel (p<0.0001).

**Figure H: Discordant amino acid changing variants per gene (both panels)**


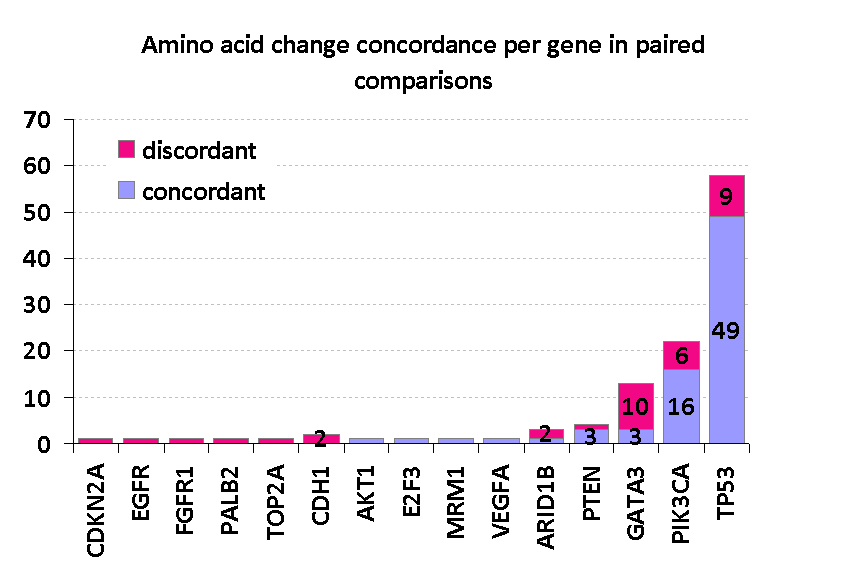


**amino acid changes per gene in paired comparisons**

For TP53 15.5% of the observed variants could not be replicated, for PIK3CA 27.3%, while 10 out of the 13 amino acid changes identified for GATA3 were discordant in the corresponding sample pairs.

**Figure I: Amplicon performance was strongly associated with variant concordance (p<0.0001)**

Discordant variant rates for grade 0, 1, 2 and 3 amplicons were 57.6%, 23.8%, 23.3% and 7.7%, respectively.
